# Sex-specific effects of developmental environment on reproductive trait expression in *Drosophila melanogaster*

**DOI:** 10.1002/ece3.243

**Published:** 2012-07

**Authors:** Dominic A Edward, Tracey Chapman

**Affiliations:** School of Biological Sciences, University of East AngliaNorwich Research Park, Norwich, Norfolk, NR4 7TJ, United Kingdom

**Keywords:** *Drosophila melanogaster*, environmental variation, genotype-by-environment interaction, larval density, larval development, sexual selection

## Abstract

Variation in the expression of reproductive traits provides the raw material upon which sexual selection can act. It is therefore important to understand how key factors such as environmental variation influence the expression of reproductive traits, as these will have a fundamental effect on the evolution of mating systems. It is also important to consider the effects of environmental variation upon reproductive traits in both sexes and to make comparisons with the environment to which the organism is adapted. In this study, we addressed these issues in a systematic study of the effect of a key environmental factor, variation in larval density, on reproductive trait expression in male and female *Drosophila melanogaster*. To do this, we compared reproductive trait expression when flies were reared under controlled conditions at eight different larval densities that covered a 20-fold range. Then, to place these results in a relevant context, we compared the results to those from flies sourced directly from stock cages. Many reproductive traits were surprisingly insensitive to variation in larval density. A notable exception was nonlinear variation in female fecundity. In contrast, we found much bigger differences in comparisons with flies from stock cages—including differences in body size, latency to mate, copulation duration, fecundity, and male share of paternity in a competitive environment. For a number of traits, even densities of 1000 larvae per vial (125 larvae per mL of food) did not phenocopy stock cage individuals. This study reveals novel patterns of sex-specific sensitivity to environmental variation that will influence the strength of sexual selection. It also illustrates the importance of comparisons with the environment to which individuals are adapted.

## Introduction

Sexual selection contributes significantly to the generation of biological diversity and its effects are manifested in diverse processes that occur both before and after mating (Andersson 1994). The strength and direction of sexual selection depends upon the level of expression of reproductive traits, and often has a strong environmental component (Emlen and Oring 1977; Shuster and Wade 2003). An important focus for research is therefore to assess the effect of key environmental conditions on the opportunity for sexual selection. This is important because it can elucidate whether sexual selection will help or hinder the adaptation of a species to novel or changing environments (Candolin and Heuschele 2008; Maan and Seehausen 2011). In general, it is realized that the influence of the environment upon sexual selection is not yet fully understood (Bussiere et al. 2008; Fricke et al. 2009; Ingleby et al. 2010).

Previous studies have tackled the effect of environmental factors on the expression of reproductive characters targeted by sexual selection. For example, the turbidity of water following eutrophication can affect the ability of female cichlid fish to choose a mate (Seehausen et al. 1997). Variation in stream flow rate has also been found to influence mate choice in the guppy (Wong and Jennions 2003), and the amount of sexual harassment experienced by female seaweed flies is influenced by the different marine algae to which males are exposed (Edward and Gilburn 2007). More generally, mate preferences (e.g., Hunt et al. 2005; Cotton et al. 2006) and sexually selected traits (e.g., Buchanan 2000; David et al. 2000) can be condition dependent, and individual condition is often strongly influenced by the environment. This can lead to genotype-by-environment interactions (Ingleby et al. 2010), that is, when the fitness of a genotype (G) depends upon the environment (E) in which it is expressed. G × E effects are widespread but their impact upon sexual selection is only just beginning to be realized (Ingleby et al. 2010).

Although environmental factors are now acknowledged to be important in sexual selection, there are important gaps remaining in our knowledge. For example, simultaneous assessments of the effects of environmental variation upon reproductive traits in both sexes are usually lacking. This is an important omission because the outcome of mating interactions is necessarily dependent upon traits expressed in both males and females. The effect of environmental variation on the likely strength of sexual selection is therefore easier to assess when both sexes have been tested under the same conditions (Ingleby et al. 2010). A second frequent omission is that only a limited range of potential environmental variation, or a limited number of levels of environmental variation, is investigated (e.g., Amitin and Pitnick 2007; Rode and Morrow 2009). An important consideration in this respect is the range of environmental variation to which the study organism is normally adapted (e.g., Jia et al. 2000). Examining the full range of ecologically relevant environmental variation allows accurate assessment of the appropriate range of potential phenotypic responses. Examining environmental variation that is beyond the range normally experienced may give mis-leading results as trait expression would not necessarily accurately reflect adaptation to these conditions. As well as the range of environmental variation, it is also important to consider the number of levels within that range that can be examined. In many cases, the environmental condition under investigation is a scalar variable from which many different levels may be examined. However, studies may often investigate a restricted set of the potential levels (e.g., just two; Amitin and Pitnick 2007; Rode and Morrow 2009). However, examining multiple levels is necessary to identify potentially important phenomena such as nonlinear effects of the environment on reproductive trait expression (e.g., Wong and Jennions 2003; Edward and Gilburn 2007; Etges et al. 2007). Also, multiple levels of environmental variation may need to be examined to refine accurately the point at which individuals are most susceptible to environmental change. A third factor of importance, we would argue, is that the effects of environmental variation are expected to have global effects on behavior and physiology, hence it is an advantage to consider a range of reproductive traits, where possible.

In this study, we aimed to conduct a systematic investigation of the effect of a wide range of environmental variation on reproductive trait expression in males and females of the fruit fly *Drosophila melanogaster*, a species that has been widely used in the study of sexual selection (e.g., Bateman 1948; Chapman et al. 1995; Rice et al. 2006; Manier et al. 2010). The stock population we used is maintained in the laboratory in large population cages with overlapping generations. Though temperature, light cycle and humidity are controlled, this population is still subject to fluctuations in population density and demography leading to likely variation in larval densities in the culture medium. Therefore, even within the relatively controlled laboratory environment, larval density is a key environmental factor predicted to have an important influence upon reproductive fitness. As it is difficult to measure larval density within the stock cages, to maintain consistency between experiments it is common practice to rear flies in smaller vials in which the larval density can be controlled. Key questions, therefore, are to what extent reproductive traits are environmentally sensitive and how reproductive trait expression varies between stock and vial cultures.

We examined the effect of a wide range of variation in larval density upon multiple reproductive traits for males and females in competitive and noncompetitive contexts. We then compared the range of trait expression found in these experiments to the same traits measured in individuals sourced directly from stock cages that served as the source of the populations examined. This study therefore provides information about how the environment can influence the expression of key target traits for sexual selection. Previous work on larval density has revealed effects on the expression of several traits. For example, flies reared at high larval density are smaller and have reduced fat content (Lazebnyi et al. 1996; Byrne and Rice 2006; Amitin and Pitnick 2007; Rode and Morrow 2009), which could underlie the significantly reduced fecundity in females (Barker and Podger 1970; Amitin and Pitnick 2007). Males reared at high larval density also have reduced reproductive success (Amitin and Pitnick 2007; Rode and Morrow 2009), which can in part be attributed to reduced sperm size and seminal receptacle length (Amitin and Pitnick 2007).

We investigated the effects of environmental variation during development (via altering larval density) on the expression of a range of different reproductive traits in both sexes of adults. First, we compared a much wider range of environmental variation than in previous studies—a total of eight increments of larval density variation, from 50 to 1000 larvae per vial (6.25 to 125 larvae per mL of food). Second, we compared a wide range of traits that can influence reproductive fitness—in addition to body size, the reproductive traits that were measured include willingness to mate, latency to mate, mating duration, egg production, fertility, and the share of paternity obtained by males in a competitive environment. Third, we compared traits expressed in both males and females. Finally, we measured the same reproductive traits in flies that were obtained directly from stock cages. This information can be used to correctly contextualize the effect of variation in larval density and also to identify the range of larval density that phenocopies the normal culture conditions to which the flies are adapted. This dataset therefore comprises a comprehensive description of the effects of resource availability upon reproduction in this species.

## Materials and Methods

### Fly rearing

Flies were sourced from the Dahomey wild-type stock population of *D. melanogaster*. This stock is maintained at large population sizes, typically in the 1000s, with overlapping generations at 25°C on a 12:12 h light:dark cycle. Standard glass vials were used throughout the experiment (75 mm × 25 mm diameter) containing 8 mL of standard sugar-yeast food (100 g brewer's yeast, 100 g sucrose, 20 g agar, 30 mL Nipagin (10% w/v solution), 3 mL propionic acid per 1 L medium).

Flies were obtained either directly from stock cages or were reared at controlled larval densities. To collect flies directly from the cages, two food bottles containing mature pupae were removed from stock cages and any adult flies were discarded. Eight hours later newly eclosed, virgin flies were collected. Flies were reared at controlled larval densities by placing a known number of first instar larvae into vials. Larval densities used were 50, 100, 200, 300, 400, 600, 800, and 1000 larvae per vial. After emergence, flies were stored in single sex vials containing 10 flies and were aged for at least 24 h to ensure sexual maturity before use in experiments. Larval development vials were each seeded with a standardized level of live yeast grains and adult vials were seeded ad libitum. The collection of flies from stock cages and controlled density vials was synchronized so that flies were of the same age at the start of the experiments.

### The effect of larval environment on male and female body size

To determine the body size of flies reared at each larval density and of flies obtained from stock cages, 25 males and 25 females from each cohort were selected at random and the right wing of each fly was removed. Wings were then mounted onto paper, photographed under a microscope, and the distance between the anterior cross-vein and wing tip was measured using ImageJ software (Abramoff et al. 2004).

### The effect of larval environment on male and female mating behavior, fecundity, and fertility

The effect of larval environment on male and female reproductive traits was assessed by recording mating behavior and offspring production in mating trials. Mating trials were repeated on four different days and all data were collected within a two-month period between July and September 2010. In each mating trial, flies that had been reared at different larval densities and flies from stock cages were mated to a reference fly that was the same age reared at a density of 100 larvae per vial. In the first set of mating trials, males that experienced different larval environments were mated to reference females. In the second set, females that experienced different larval environments were mated to reference males.

In each mating trial, one male and one female were placed in a standard vial containing 8 mL of food and a small drop of yeast paste. Flies were observed for a maximum of 2 h or until mating occurred. The latency to mate and copulation duration were recorded for each mating. Immediately after mating males were removed from vials. Females remained in vials for a further 24 h after which they were also removed and the number of eggs laid was counted. Vials were retained for 12 days to allow offspring to develop. The number of adult offspring that eclosed following each mating trial was then used to determine the proportion of eggs that were fertile. The data accumulated for each pair of flies therefore comprised the occurrence of mating, latency to mate, copulation duration, egg production, and fertility.

### The effect of larval environment on male share of paternity in a competitive environment

To test the effect of larval environment on male competitive ability, males reared at different larval densities and from stock cages were set up to compete with a competitor male for a share of paternity. Males were placed into individual vials with one female and one competitor male. All females and competitor males were reared at a density of 100 larvae per vial and carried a recessive *sparkling^poliert^* eye color mutation so that the paternity of offspring could be accurately attributed to each male. Flies were transferred to fresh food daily for three days and all vials were retained for 12 days to allow development of offspring. The proportion of offspring sired by the focal male was used as a measure of competitive ability. This competitive environment provided a combined measurement of a male's ability to compete against other males in both courtship prior to mating and in post-copulatory sperm completion.

### Statistical analysis

Body size, the proportion of flies that mated, latency to mate, copulation duration, egg production, fertility, and male share of paternity in a competitive environment were each analyzed in separate general linear models for males and females. Normal, binomial, and quasibinomial error distributions were used as appropriate. For each trait, two models were analyzed. The first model considered differences between flies when reared in vials at different larval densities. These models included a fixed effect of larval density. The second model considered differences between flies reared in vials and flies from stock cages. If there was no significant effect of larval density in the first model, then flies from all larval densities were pooled to form a single treatment that was compared to flies from stock cages. If there was a significant effect of larval density in the first model, then flies from stock cages were instead compared to flies reared at a standard larval density whose mean trait value most closely matched that found in stock cages. In addition to fixed effects of larval environment, each model also included a fixed effect of replicate and its interaction with larval environment. To account for inequality of variance in body size between vials and stock cages, data in these models were weighted by the inverse of the variance for each group. Latency to mate was log transformed to approximate a normal distribution.

For each model, the significance of fixed effects was compared in an analysis of deviance, and nonsignificant effects were removed when this did not significantly influence the fit of the model at *P* = 0.05. To perform contrasts of the effect of larval density, factor levels were compared in ascending order. Helmert contrasts, which compare each factor level to the mean of all preceding factor levels, were used to look for trends between different larval densities.

Levene's tests for equality of variance were used to determine if there were any significant differences in variance in latency to mate, mating duration, egg production, and body size between larval environments. These tests were conducted separately for each sex and included stock cage as a factor level alongside each larval density. All analyses were performed using R v. 2.11.1.

## Results

### The effect of larval environment on body size

**Figure 1:**
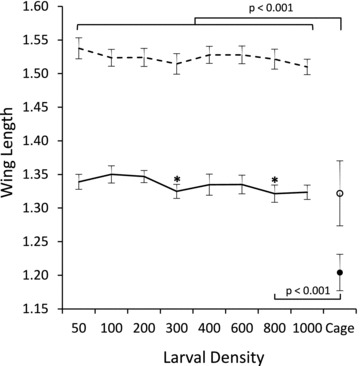
The wing length of males (solid line/solid circle) and females (dashed line/open circle) from individuals reared at larval densities of 50 through to 1000 larvae per vial and from individuals reared in stock cages (mean in mm ± 95% confidence interval). A total of 25 males and 25 females from each condition were selected at random and the right wing of each fly was removed. The distance between the anterior cross vein and wing tip was measured.

Significant sexual size dimorphism was found, with females being significantly larger than males (*F*_1,434_ = 3274.1, *P* < 0.001; Fig. 1). Male, but not female, body size varied significantly with larval density; however, this effect was small compared to a much greater reduction in size for both sexes that developed in stock cages (Table 1; Fig. 1). Female size appeared more sensitive to the larval environment than male size (Fig. 1). A conservative estimate of this difference in sensitivity is found by comparing larval densities that produced the smallest males (800 larvae per vial) and the smallest females (1000 larvae per vial) to male and female size when larvae developed in stock cages. Males reared in stock cages had a wing length that was 0.13 mm shorter (a reduction of 9.8%) compared to males reared at 800 larvae per vial. Females reared in stock cages had a wing length that was 0.20 mm shorter (a reduction of 13.2%), compared to females reared at 1000 larvae per vial. A significant interaction between sex and larval environment significantly influenced body size (*F*_1,92_ = 6.104, *P* = 0.015). Variance in body size was also found to differ significantly between larval environments (Table 2) as both male and female body size was more variable when larvae developed in stock cages than in vials (Fig. 1).

**Table 1:** Results of general linear models examining the effect of larval density on reproductive traits in males and females. The error distribution used in each model is indicated in parentheses. Significant effects are highlighted in bold

	Male larval environment varied		Female larval environment varied	
		
	Variation between standard density vials	Variation between vials and cages	Variation between standard density vials	Variation between vials and cages
Body size (normal)	*F*_7,190_ = 2.967	*F*_1,45_ = 64.645	*F*_7,191_ = 1.492	*F*_1,222_ = 66.056
	***P* = 0.006**	***P* < 0.001**	*P* = 0.172	***P* < 0.001**
Proportion mating (binomial)	X^2^_7_ = 5.018	X^2^_1_ = 0.383	X^2^_7_ = 2.799	X^2^_1_ = 0.371
	*P* = 0.658	*P* = 0.536	*P* = 0.903	*P* = 0.371
Latency to mate (normal)	*F*_7,367_ = 1.307	*F*_1,429_ = 5.413	*F*_7,362_ = 1.621	*F*_1,421_ = 5.135
	*P* = 0.246	***P* = 0.020**	*P* = 0.128	***P* = 0.024**
Mating duration (normal)	*F*_7,361_ = 0.326	*F*_1,423_ = 20.345	*F*_7,364_ = 1.904	*F*_1,420_ = 0.041
	*P* = 0.942	***P* < 0.001**	*P* = 0.068	*P* = 0.839
Egg production (normal)	*F*_7,364_ = 1.752	*F*_1,426_ = 0.479	*F*_7,365_ = 10.303	*F*_1,84_ = 12.130
	*P* = 0.096	*P* = 0.489	***P* < 0.001**	***P* < 0.001**
Fertility (quasibinomial)	*F*_7,354_ = 0.759	*F*_1,415_ = 0.453	*F*_7,356_ = 0.702	*F*_1,413_ = 0.655
	*P* = 0.622	*P* = 0.501	*P* = 0.671	*P* = 0.655
Share of paternity in competitive	*F*_7,267_ = 2.987	*F*_1,68_ = 21.742	-	-
environment (quasibinomial)	***P* = 0.005**	***P* < 0.001**		

**Table 2:** Levene's test for the equality of variance between different larval density treatments for latency to mate, mating duration, egg production, and body size. Significant deviations from equal variance are highlighted in bold

	Male larval density varied	Female larval density varied
Body size	*F*_8,211_ = 3.126, ***P* = 0.002**	*F*_8,215_ = 12.731, ***P* < 0.001**
Latency to mate	*F*_8,419_ = 0.663, *P* = 0.724	*F*_8,417_ = 0.401, *P* = 0.920
Mating duration	*F*_8,419_ = 1.108, *P* = 0.356	*F*_8,417_ = 1.228, *P* = 0.281
Egg production	*F*_8,419_ = 0.792, *P* = 0.610	*F*_8,417_ = 1.353, *P* = 0.215

### The effect of larval environment on male and female mating behavior, fecundity, and fertility

**Figure 2:**
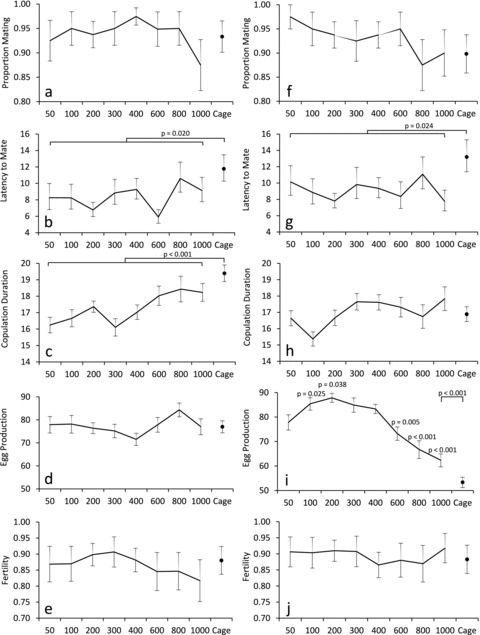
Behavioral and physiological traits measured in the mating behavior, fecundity, and fertility assays (mean ± 95% confidence intervals) for males and females reared at larval densities of 50 through to 1000 larvae per vial and reared in stock cages. Panels (a–e) on the left show trait values for density treatment males held with reference females. Panels (f–j) on the right show trait values for density treatment females held with reference males. We recorded the proportion of individuals mating, latency to mating, duration of mating, number of eggs laid in the 24 h after mating, and the fertility of eggs laid in the 24 h after mating.

A total of 917 mating trials were completed. The majority of male and female traits that were measured were unaffected by variation in larval density in vials (Table 1; Fig. 2). The only trait affected by larval density was egg production in females raised at different densities. Female fecundity followed a nonlinear pattern, significantly increasing as larval density increased from 50 to 200 larvae per vial but then declining at densities above 400 larvae per vial (Fig. 2i).

In contrast, many of the traits that were measured did vary when comparing flies that developed in vials to flies that developed in stock cages (Table 1). There was no difference in the proportion of flies that mated (Fig. 1a and f); however, both males and females that developed in stock cages took longer to mate than their counterparts that developed in vials (Fig. 1b and g). There were also significant sex-specific effects. Mating duration was increased when males, but not females, developed in stock cages (Fig. 1c and h). Egg production was decreased when females, but not males, developed in stock cages (Fig. 1d and i). Fertility was unaffected by the larval environment of males or females (Fig. 1e and j). There was no significant heterogeneity of variance for any of the traits examined (Table 2).

### The effect of larval environment on male share of paternity in a competitive environment

**Figure 3:**
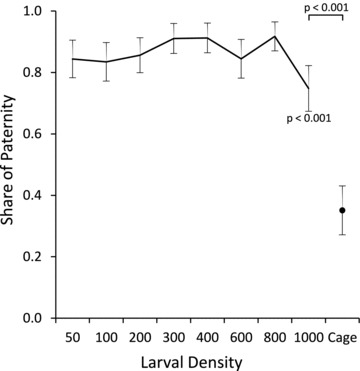
The share of paternity achieved by males reared at different larval densities and from stock cages (mean share of paternity ± SE). To measure a male's share of paternity achieved under competitive conditions, males were placed into individual vials with one female and one competitor male for three days and the number and paternity of offspring determined.

A total of 311 males were assayed for their ability to obtain paternity when competing with another male (mean sample size per larval environment = 34.6, range = 32–38). A male's share of paternity was significantly reduced at high larval density, but this influence was only evident when reared at 1000 larvae per vial (Table 1; Fig. 3). There was a much greater reduction in male share of paternity following development in stock cages when compared to development in vials (Table 1; Fig. 3).

## Discussion

In this study, we investigated the effect of variation in larval environment upon the expression of a range of traits influencing reproductive fitness in both males and females. We first compared traits when flies were reared in vials across a wide range of larval densities. We then contrasted trait values following development in vials with measurements of flies taken directly from the stock cage environment.

### The effect of larval density on expression of reproductive traits in males and females

The first main finding was that the majority of reproductive traits were unaffected by variation in larval density as manipulated in vials (Table 1; Fig. 2). Male body size did decrease at higher densities, but this effect was small in comparison to the difference in male body size between vial and stock cage environments (Fig. 1). The traits most significantly affected by larval density were female fecundity (Fig. 1i) and male share of paternity in a competitive environment (Fig. 3). The nonlinear influence of larval density on female fecundity is a novel finding and indicates potential facilitation between larvae during development, as has been reported in other insect species (e.g., Leggett et al. 1996; Fletcher 2009; Ronnas et al. 2010).

This finding that most traits were unaffected by larval density was unexpected and is in contrast to previous studies that have found effects of larval density on reproductive traits (Byrne and Rice 2006; Amitin and Pitnick 2007; Rode and Morrow 2009). Larval density is expected to limit the amount of food resources available to each individual and therefore the ability to express reproductive traits. One possibility is that food was not limiting within the range of densities tested in this study. However, the observed environmental sensitivity of at least some traits, in particular female fecundity, argues against this interpretation. In addition, the range of larval densities used in this study is greater than in previous studies that have found an effect of larval density (Amitin and Pitnick 2007; Rode and Morrow 2009). A more plausible suggestion is that the pattern of environmental insensitivity in some traits resulted from life-history trade-offs between current reproductive trait expression, future reproductive trait expression, and somatic maintenance. For example, zebra finches *(Taeniopygia guttata*) maintain the expression of reproductive traits when reared on a poor diet, but only at a cost to future longevity (Birkhead et al. 1999). Such trade-offs would not have been identified with the methodology used in this study. Hence, life histories as a whole should be considered in order to accurately quantify environmental effects on reproductive success and the opportunity for sexual selection (e.g., Edward et al. 2011).

### Comparison of vial and stock cage environments

Though only a few traits varied significantly in response to differences in larval density, many of the other traits measured—body size, latency to mate, mating duration, egg production, and share of paternity—were significantly different when one or both sexes developed in stock cages instead of vials (Table 1). Flies reared in stock cages were significantly smaller than flies reared in vials and, because of greater female sensitivity to the larval environment, sexual size dimorphism was also significantly reduced among cage-reared flies (Fig. 1). Males and females that developed in stock cages took significantly longer to mate than flies reared in vials. One explanation is that male *D. melanogaster* prefer larger, more fecund females (Byrne and Rice 2006) so were less willing to mate with smaller females from stock cages. Similarly, larger male *D. melanogaster* are known to deliver more courtship to females (Partridge et al. 1987), which could explain the shorter latency to mate of larger males reared in vials.

Copulation duration was significantly longer when males were reared in stock cages. It could be that smaller males need to copulate for longer to achieve a level of reproductive fitness similar to that of larger males (e.g., Parker and Simmons 1994). In the context of a single mating opportunity, we found no difference in the reproductive fitnesses of males reared in cages or vials as the egg production and fertility of females was the same (Fig. 2d and e). This indicates that the longer copulation duration of stock cage flies was sufficient to offset any costs resulting from their reduced body size. However, within a more realistic, competitive environment smaller males that had developed in stock cages were at a significant disadvantage (Fig. 3). This is consistent with previous reports of an association between male size and reproductive success in *D. melanogaster* (Bangham et al. 2002).

A key finding was that for all of the traits that showed sensitivity to larval conditions, flies taken from stock cages showed the most extreme responses. Hence, we did not phenocopy reproductive trait expression for either sex even at 1000 larvae per 8 mL food. This was unexpected, but gives insight into the relatively harsh environmental conditions to which the flies are adapted in stock cages. The effective larval density experienced by flies in the stock cages could exceed 1000 larvae per vial. However, there are several factors other than larval density that differ. For example, cages comprise overlapping generations and competition with older and larger larvae present in stock cultures could be more severe than competition within a cohort of similar age. Adults are also present during larval development in stock cages. It is possible that larvae can detect the presence of adults, which could influence their development in response to perceived levels of competition (e.g., Gage 1995). Alternatively, waste products from large numbers of adults could differentially influence the quality of food. The total volume of food present also differs and smaller volumes of food in vials may contribute to greater edge effects and desiccation. In contrast, even though there is a greater depth of food in bottles, the deeper parts are not accessed by larvae (D. A. Edward and T. Chapman, pers. obs.). Overall, vial environments are comparatively benign in comparison to stock cages. This result is significant for studies that use variation in larval density to study environmental interactions and sexual selection.

### Sex-specific environmental sensitivity

The results also provide insight into sex-specific differences in sensitivity to environmental change. For example, a greater sensitivity of female body size to developmental conditions in the stock cages reduced the extent of sexual size dimorphism in comparison to the controlled density treatments (Fig. 1). The reproductive traits most sensitive to variation in larval density were female fecundity (Fig. 2i) and male success in a competitive environment (Fig. 3). However, the pattern of sensitivity differed for each sex. There was a more abrupt reduction in male fitness at densities above 800 larvae per vial that contrasted markedly with the initial increased followed by a more progressive decline in female fecundity under these conditions (Figs. 1i and 3). Sex-specific sensitivity to developmental environment has been identified in a previous study, which showed that female seminal receptacle length is more sensitive than sperm length to the developmental environment (Amitin and Pitnick 2007). Hence overall, we conclude that there is good evidence for sex-specific sensitivity in reproductive trait expression; however, there is no consistent evidence that one sex appears consistently more sensitive than the other.

Sex-specific sensitivity to environmental variation may also be indicative of sex-specific variation in life-history trade-offs. An example is found in the caddisfly (*Agrypnia deflate*), in which the expression of reproductive traits in females is more plastic in response to larval nutrition than is found for male life-history traits (Jannot et al. 2007). An important consideration for male *D. melanogaster* is potential trade-offs between pre- and postcopulatory reproductive traits following resource limitation. Here, variation in male success in a competitive environment (Fig. 3) was a measurement of the combined expression of both pre- and postcopulatory traits. However, as the proportion of males mating (Fig. 2a) and the latency to mate (Fig. 2b) were not significantly affected by the larval environment, the reduction in male success in a competitive environment is more likely to have resulted from changes in postcopulatory trait expression, that is, traits that influence success during sperm competition. This prediction is consistent with previous studies of *D. melanogaster* that have found significant effects of larval density on postcopulatory male reproductive traits (Amitin and Pitnick 2007; Rode and Morrow 2009). Life-history trade-offs that favor the preservation of pre- over postcopulatory traits would be predicted if precopulatory mating traits are more strongly linked to male reproductive success.

### Contrasts with previous studies

Contrasts between our results and those of related previous work may lie in differences in culturing and experimental design. For example, Rode and Morrow (2009) used flies from the LH_M_ stock population, which is routinely reared in vials at controlled densities of between 150 and 200 larvae per 10 mL of food. Their experimental treatments were 400 eggs in 10 or 1 mL of food. Variation in larval density is not controlled within our Dahomey stock population, which allows a greater opportunity for evolutionary adaptation to variation in larval density. This could explain why, at similar densities, Rode and Morrow (2009) found a reduction in male reproductive fitness under competitive conditions and we did not. This highlights the need to consider different stock husbandry practices when studying the effect of variable environmental conditions. Amitin and Pitnick (2007) used flies maintained under similar conditions as in the Dahomey stock (uncontrolled larval densities). A significant effect of larval density (at 15, 75, and 200 larvae per 8 mL of food) on sperm and seminal receptacle length was found (Amitin and Pitnick 2007). Our results suggest that these traits would likely show even more marked differences in adults taken directly from stock cages.

## Conclusion

In this study, we identified sex-specific effects of varying larval density for a number of reproductive traits. In contrast to predictions, the majority of traits were insensitive to this manipulation. However, by assessing a comprehensive range of variation, we identified nonlinear effects of larval density on female fecundity. A further important finding is that an effect of larval density on male reproductive fitness was only evident within a competitive environment. This work shows that factors other than larval density are also important within cages, in influencing reproductive trait expression.
